# Mucormycosis and COVID-19 an epidemic in a pandemic?

**DOI:** 10.3126/nje.v11i2.37342

**Published:** 2021-06-30

**Authors:** Indrajit Banerjee, Jared Robinson, Mohammad Asim, Brijesh Sathian, Indraneel Banerjee

**Affiliations:** 1-2 Sir Seewoosagur Ramgoolam Medical College, Belle Rive, Mauritius; 3 Trauma Surgery, Hamad General Hospital, Doha, Qatar; 4 Geriatric and long term care Department, Rumailah Hospital, Hamad Medical Corporation, Doha, Qatar; 5 Uro oncologist and Robotic Surgeon, Apollo multi speciality Hospitals, Kolkata, West Bengal, India

**Keywords:** COVID-19, India, Mycoses, SARS-CoV-2, SARS-CoV-2 delta variant, SARS-CoV-2 variants, Zygomycosis

## Abstract

Mucormycosis and aspergillosis are rare, invasive and life-threatening infections primarily caused by Rhizopus arrhizus and Aspergillus fumigatus with higher case fatality rates (>50%), respectively. Invasive Aspergillosis and Mucormycosis have been established and recognized as complications of the SARS-CoV-2 infection. Such cases have been intimately linked and related to prior corticosteroid therapy. With the new highly infectious Delta strain (B.1.617.2 and B.1.617.2.1 or AY.1) of the coronavirus which is running rampant throughout India causing unprecedented death tolls, a new crisis is evolving. Invasive “black fungus” (Mucormycosis) is creating an epidemic within a global pandemic. The unique socio-economic, genetic and health status of Indian population culminates into a melting pot which sustains the viable triad for the “black fungus” infection to gain a stronghold. Diabetes mellitus, immunosuppression and the current COVID-19 global pandemic with its massive surges in the country have produced the “perfect storm.” Ophthalmologist across India have reported a surge in invasive Mucormycosis cases with a rise in orbital compartment syndrome often calling for radical procedures such as enucleation surgeries. The “black fungus” pandemic and invasive Mucormycosis resulted in the sinister secondary infections and complications are closely linked with the COVID-19 infection in India. It is therefore of the upmost importance that neighbouring countries particularly Nepal and other Asiatic nations take great cognizance of this indolent “black fungus killer” and ensure new screening and testing protocols for early identification to ensure effective management.

## Background

The rapid and uncontrollable spread of COVID-19 has led to an unprecedented time in the 21st century. The sheer novelty, lack of understanding of disease pathophysiology and the absence of established treatment regimen and protocol have garnished the global race setting to discover the most suitable drug and treatment regimen. In doing so, the selection of potential drugs and therapies to treat COVID-19 patients varied from antiviral therapy to antiprotozoal drugs at different institutions [1].

The use of a wide range of therapies with different dose regimes in individuals with multiple and varied comorbidities, resulted in the mixed outcome and adverse drug effects, which could be catastrophic. This situation arises in India with an increase in Mucormycosis and orbital compartment syndrome in patients with COVID-19 [2].

### Mucormycosis and aspergillosis

Mucormycosis and aspergillosis are rare, invasive and life-threatening infections. The causative agents being namely: Aspergillus fumigatus and Rhizopus arrhizus. The case fatality rates being over 50%, respectively [3].

### Predisposition to infection

Invasive fungal infections are usually isolated in patients who suffer from severe immunocompromised states due to comorbidities such as diabetes mellitus, uremia, both solid organ and hematological malignancies and chiefly corticosteroid therapy. In India, however, there is a greater likelihood of immunocompetent patients contracting such mucormycotic infections [3]. Uncontrolled hyperglycemia, excessive use of corticosteroids for immunosuppression, and long-term admissions in the critical care unit are the most common causes of mucormycosis in COVID-19 patients [4]. Mucormycosis is more likely to develop in organ transplant recipients due to persistent immunosuppression and concomitant diseases like diabetes. As a result, COVID19 transplant recipients must undergo a complete clinical evaluation to rule out mucormycosis [5].

Rhizopus arrhizus infections predominantly involve the rhino-orbital-cerebral, pulmonary syndromes, cutaneous and gastrointestinal system. These infections are highly angio-invasive and may spread in severely immunocompromised patients, leading to high morbidity and mortality rates [2,3].

Aspergillus fumigatus predominantly cause Aspergillosis infections, and the conditions are intimately related to patients on immunosuppressive-corticosteroid therapy [2,6].

### COVID-19 and invasive mycosis

Invasive Aspergillosis and Mucormycosis have been established and recognized as a complication of the SARS-CoV-2 infection. These infections are present in severely ill patients whom are undergoing treatment in the intensive care unit (ICU). It is noted that close to 35% of these COVID-19 patients in ICU have contracted invasive pulmonary aspergillosis. These cases have been closely linked to prior corticosteroid therapy [6,7].

### Epidemiology of COVID-19 and concomitant mycosis

Globally, the reported burden of mucormycotic cases (71% of the global cases) is highest in India. In contrast, there are reports of sporadic cases of Mucormycosis intimately linked to COVID-19 infections in other parts of the world. For example, a study published by Chen N, et al., who preformed fungal cultures on 99 COVID-19 positive patients; discovered that 5 out of the 99 cultures were positive 5.1%. The infectious agents being namely aspergillus flavus and Candida albicans [8]. A similar study performed by Yang, et al. diagnosed 3 out of 52 of the patients tested (nearly 6%) with concomitant mycotic infections [9].

In the European region, similar events are unfolding with a study in the Netherlands reported that out of 31 tests being conducted in ICU settings; 5 had confirmed diagnosis of concomitant Aspergillus fumigatus infections, this equating to a higher outcome infection ratio with a percentage of nearly 20% [10]. A more flawed picture is reported in Germany, which states that invasive pulmonary aspergillosis (IPA) was diagnosed in 26% of a consecutive cluster of 19 patients who suffered from severe COVID-19 infections [11].

A case series of the histopathological findings of autopsy in 10 patients who had succumbed to COVID-19 and its complications in the United Kingdom revealed: signs of thrombosis in one of the major organs in all of the autopsies, the most prevalent sign being diffuse alveolar damage in each of the ten patients. Compared to India, disseminated Mucormycosis was only isolated in 1 of the 10 patients (accounting for 10%) [12].

A case report from the Gastrointestinal Endoscopy Unit in Brasil, reported a case of Gastrointestinal Mucormycosis more specifically zygomycosis in an 86-year-old male COVID-19 positive patient. The patient presented to the emergency room with a haemoglobin of 14.3 mg/dL, five days after that, developed melena and recorded a haemoglobin level of 5.6 mg/dL. The patient was subsequently transfused, and an esophagogastroduodenoscopy was performed. An esophagogastroduodenoscopy revealed that the patient was found to have two large gastric ulcers with deep haemorrhagic bases. Biopsies of the ulcers were cultured, and mucormycosis was found to be the causative agent. The patient succumbed to the infection 36 hours subsequent to the endoscopy [13].

In a prospective observational clinical study conducted in Turkey, 11 incidences of microbiologically proven rhino-orbital mucormycosis were discovered among 32,814 patients hospitalized with COVID-19. Due to immunological dysregulation and extensive use of steroids, severe COVID-19 has been linked to a considerable incidence of rhino-orbital mucormycosis and higher mortality rates [14]. Fifteen cases of rhino-orbital mucormycosis and COVID-19 were reported in another cross-sectional descriptive multicenter study from Iran. Diabetes mellitus with poor control is a major risk factor for COVID-19 associated mucormycotic cases (CAM) [15].

### “Black fungus” crisis in India

With the new strain of the coronavirus (B.1.617.2 and B.1.617.2.1 or AY.1) running rampant throughout India, causing unprecedented death tolls, a recent crisis is evolving. Invasive “black fungus” (Mucormycosis) is creating an epidemic within a global pandemic. The unique socio-economic, genetic and health status of Indian population culminates into a melting pot that sustains the viable triad for the “black fungus” infection to gain a stronghold. Diabetes mellitus, immunosuppression and the COVID-19 pandemic with its massive surges in the country produce the “perfect storm.” In Maharashtra alone, the health minister has reported that there are believed to be no less than 2000 cases of Mucormycosis in the state alone [6].

In the city of Gujarat, 40 such cases have been reported, with 8 patients had undergone complete enucleation surgeries with massive further dissection and wound debridement. The majority of the cases have been reported to have occurred in diabetic patients who have recovered from the initial COVID-19 infection. In addition, eye surgeons from around India are registering a surge in invasive mucormycosis cases with a rise in orbital compartment syndrome, often calling for radical procedures such as enucleation surgeries [16].

A study by Singh A, et al. reported 101 cases of Mucormycosis associated with COVID-19. Eighty-two (81%) of the total cases being recorded from India. The most common site for infection being the nose and sinuses 88.9%. The Mucormycosis infections were predominantly found in males (78.9%) as opposed to females (21.1%). These cases were identified in both individuals who had recovered from the COVID-19 infection (40.6%) as well as in active cases (59.4%). It is stipulated that diabetes mellitus is present in 80% cases and corticosteroid therapy was given in 76.3% of the individuals suffering from COVID-19. The overall mortality was reported to be 30.7% [16].

A systematic review undertaken by Pal R, et al. reveals that out of the 99 CAM cases published to date, 72% of them were of Indian origin. Males were found to be more affected with 78% of all the patients being male in gender. The most prevalent comorbidity present in 85% of these patients was diabetes mellitus. 37% of the patients contracted the mucormycotic infection after their initial recovery from COVID-19. A median interval of 15 days has been reported between the initial diagnosis of COVID-19 and the apparence of first signs of mucormycotic infections. The most common form of Mucormycosis reported in 42% of the patients was that of the rhino-orbital type. Corticosteroid therapy (85%) was mainly used for treatment [17]. In a retrospective multicenter study conducted in India between September and December 2020, it was discovered that 187 (65.2%) of 287 patients with mucormycosis had CAM, with a prevalence of 0.27 percent among hospitalized COVID-19 patients. A 2.1-fold rise in mucormycosis was detected when comparing the study period to the same period in 2019 [18]. As a result, physicians should be aware of the possibility of invasive secondary fungal infections in patients with COVID-19 infection, particularly in those who have prior risk factors, and should be able to detect and treat these infections early, reducing mortality and morbidity [19]. The states of Gujarat 22% and Maharashtra 21% had the highest prevalence of rhino-orbital-cerebral mucormycosis, according to a retrospective observational study of 2826 patients [20].

The disproportionatly higher rate of Mucormycosis in India has caused many a debate among physicians with two main schools of thought being born. One school of thought hypothesized the root cause to be the overuse of corticosteroids and immunosuppressive therapy to treat COVID-19 patients, whereas the other fervently declares that the patients with underlying metabolic syndrome coupled with the use of traditional medicine such as cow dung and urine approximate the invasive infection [7, 21].

### Mucormycosis crisis in a global context

Due to the innate complications faced by India in light of its socio-geographic and population size a true accurate population-based census is near impossible to obtain. However, it has been estimated that the high prevalence of Mucormycosis in India is nearly 70 times that of the global norm and mean. Currently, in India diabetes mellitus is the most prevalent underlying comorbidity associated with Mucormycosis followed by haematological neoplasms or malignancy, and solid organ transplantation. It has been found that patients suffering from kidney diseases (namely chronic kidney diseases in the form of renal failure) as well as pulmonary tuberculosis (namely post pulmonary tuberculosis) are at a higher risk for contracting mucormycotic infections. The most prevalent causative agent of Mucormycosis in India is Rhizopus arrhizus; however other etiological agents causing such mucormycotic infections are being noted. Such notable infections are being caused by Rhizopus homothallicus, R. microsporus and Apophysomyces variabilis [22].

### Laboratory diagnosis of invasive mycosis

A key facet in the treatment and subsequent outcome of patients suffering from invasive Aspergillosis and Mucormycosis is the early detection of infections. The infection often begin in an indolent manner and are thus only detected at an advanced stage in a patient who is already crippled in their immunological capabilities. The specimen collection varies depending on the invasion site; however, for the COVID-19 cases, the mainstay specimen collection is via bronchioalveolar lavage. Subsequently, the tissue samples, blood and sputum can be used for culture [3]. Histology and tissue culture are used to make a diagnosis, which can be intrusive, slow, and insensitive. There is a scarcity of serology tests or serum biomarkers to aid in early diagnosis. Molecular approaches, on the other hand, are being developed [23]. Newer novel methods have been developed for the detection of invasive aspergillosis such as the (LFD) Aspergillus-specific lateral-flow assay tests, which are used alongside a galactomannan enzyme-immunoassay [3,24]. As a result, it is prudent to evaluate the risk factors, kinds of invasive mycosis, diagnostic method strengths and limits, clinical situations, and the need for standard or tailored treatment in COVID-19 patients. We present a clinical flow diagram to aid physicians and laboratory experts in the management of COVID-19 patients with aspergillosis, candidiasis, mucormycosis, or cryptococcosis as co-morbidities [25]. The European Confederation for Medical Mycology and the International Society for Human and Animal Mycology formed a working group to develop consensus criteria for a case definition of COVID-19-associated invasive pulmonary aspergillosis and to provide current management recommendations for the diagnosis and treatment of such patients. Researchers can use three separate grades (possible, probable, and verified CAPA) to uniformly identify patients in registries and interventional clinical trials [26]. Furthermore, the National Task Force on COVID-19 of the Indian Council of Medical Research has created an evidence-based advice for Mucormycosis screening, diagnosis, and management [27].

### Treatment of invasive mycosis

The early diagnosis and subsequent treatment of invasive mycosis is vital for a favourable for better outcome and prognosis. The poor outcome of such infections is partly due to the severe invasiveness and hardiness of the mycotic infections and the severe immunocompromised states in the patients in which such infections occur.

The general holistic treatment is encompassed by radical debridement and excision of the infected tissues with a concomitant high-dose therapy of amphotericin B (an antimicrobial based antifungal). The first line of treatment for aspergillosis is either Voriconazole or Isavuconazole [3,28]. The current guidelines or recommendations on this subject are based on poor-quality evidence. In severe or serious COVID-19 patients, however, empirical antibiotic therapy may be recommended. Right now, the ideal strategy might be to utilize antimicrobial drugs appropriately in accordance with an antibiotic stewardship program [29].

### Corticosteroid therapy and concomitant co-mycotic infections

In particular, the use of high-dose corticosteroid therapy, in particular long-acting potent species such as Dexamethasone, has been praised for its permissive actions in the treatment of the SARS-CoV-2 infection. The underlying mechanism of action being immunosuppressive in nature to minimize the deleterious effects of the cytokine storm caused by the infection, thus often acting as a lifesaving drug in such viral infections. However, the use of such corticosteroid therapy is double-edged in nature as most COVID-19 patients have underlying concomitant comorbidities and in India diabetes mellitus is the most prevalent comorbidity. In normal circumstances, the use of high dose corticosteroid therapy in patients suffering from diabetes mellitus is contraindicated, however in patients with both diabetes mellitus and the superimposed SARS-CoV-2 infection, the use of such corticosteroid therapy is lifesaving and thus warranted. On the other hand, corticosteroid therapy may worsen the diabetic control, thus creating the perfect environment for such opportunistic mucormycotic infections. Added to the negative effect that corticosteroids have on diabetic control another large drawback is the immunosuppressant nature of the drug which gives infections such as Mucormycosis another foothold for invasion [30-32]. Glycaemic control, discontinuation of corticosteroid therapy, thorough surgical debridement, and antifungal treatment are the current management recommendations [33].

## Conclusion

The current COVID-19 situation is evolving rapidly, with no alternatives to current treatment regimens, the use of immunosuppressive therapy will remain chief in the arsenal of medicaments used by physicians. It is therefore of paramount importance that patients with pre-existing immunosuppressive diseases as well as patients with severe COVID-19 infections are screened thoroughly for any forms of invasive Mucormycosis to initiate early antimycotic treatment and retard the spread of the fungus. The “black fungus” pandemic and invasive Mucormycosis occurring in COVID-19 patients in India are a depiction of the sinister secondary infections and complications intimately linked with the virus. It is therefore of the utmost importance that neighbouring countries of India particularly Nepal and other Asiatic nations, take great cognizance of this indolent “black fungus killer” and ensure new screening and testing protocols are put into place. For prevention, it is also vital to educate patients in order to break various cultural practices that may increase the likelihood of acquiring and contracting the mycotic infection. Therefore, early screening and testing protocols are imperative. The implementation and use thereof with greater patient education will be the best tool to prevent an increase in life-threatening mucormycotic infections.
